# Determination of Knee Joint Line Positioning by Femoral Bicondylar and Epicondylar Distances in the Brazilian Population

**DOI:** 10.1055/s-0045-1811628

**Published:** 2025-11-04

**Authors:** Leonardo Augusto Melo de Andrade, Márcio de Castro Ferreira, Carlos Eduardo da Siveira Franciozi, Enzo Salviato Mameri, Marcelo Seiji Kubota, Marcus Vinícius Malheiros Luzo

**Affiliations:** 1 Universidade Federal de São Paulo, São Paulo, SP, Brazil; 2 Knee Group Surgery, Department of Orthopedics and Traumatology, Escola Paulista de Medicina, Universidade Federal de São Paulo, São Paulo, SP, Brazil; 3 HCor, São Paulo, SP, Brazil

**Keywords:** arthroplasty, replacement, knee, knee prosthesis, osteoarthritis, orthopedic procedures, prostheses and implants, artroplastia do joelho, osteoartrite, procedimentos ortopédicos, prótese de joelho, próteses e implantes

## Abstract

**Objective:**

To determine the positioning of the knee joint line height based on anatomical references, that is, the distance of the femoral condylar bicortical axis and the medial and lateral epicondylar distances in the Brazilian population.

**Methods:**

We analyzed 500 magnetic resonance imaging tests of the knees of 250 women and 250 men to measure the condylar bicortical axis (CBA) and the distance of the joint height to the medial epicondyle (MED) and to the lateral epicondyle (LED).

**Results:**

The mean age of the patients was 50.91 years old, with a standard deviation (SD) of ± 14.76. The mean CBA distance was 72.11 ± 5.93 mm. The mean MED and LED values were 33.39 ± 3.50 mm and 26.32 ± 4.08 mm, respectively. The formulas to estimate the distance of the joint line from the medial and lateral epicondyles were MED = 0.4618 x CBA and LED = 0.3615 x CBA for male subjects and MED = 0.4653 x CBA and LED = 0.3767 x CBA for female subjects using a 95% confidence interval.

**Conclusion:**

The distance to the femoral CBA can be a reference for determining the joint line positioning from the distances of the medial and lateral femoral epicondyles.

## Resumo

**Objetivo:**

Determinar o posicionamento da altura da linha articular de joelhos a partir das referências anatômicas da distância do eixo bicortical condilar femoral e das distâncias epicondilares medial e lateral na população brasileira.

**Métodos:**

Foram analisados 500 exames de ressonância magnética de joelhos de 250 mulheres e 250 homens submetidos às mensurações das medidas do eixo bicortical condilar (EBC), da distância da altura articular do epicôndilo medial (DEM) e da distância do epicôndilo lateral (DEL).

**Resultados:**

A média de idade dos pacientes analisados foi de 50,91 ± 14,76 anos. A distância média do EBC foi 72,11 ± 5,93 mm. As DEM e DEL médias foram 33,39 ± 3,50 mm e 26,32 ± 4,08 mm. As fórmulas encontradas para estimar a distância da linha articular a partir do epicôndilo medial e lateral nos homens foram DEM = 0,4618 x EBC e DEL = 0,3615 x EBC e, nas mulheres, DEM = 0,4653 x EBC e DEL = 0,3767 x EBC para intervalo de confiança de 95%.

**Conclusão:**

A distância do EBC femoral pode ser usada como referência para a determinação do posicionamento da linha articular a partir das distâncias dos epicôndilos femorais medial e lateral.

## Introduction

Total knee arthroplasty (TKA) is the gold standard surgery for gonarthrosis treatment in patients who do not respond to drug and physical therapy. It is a growing procedure worldwide. 1 2 As the number of primary TKAs increases, so do TKA revision rates. In the United States, TKA revisions account for ∼ 10% of primary arthroplasties. 1

Total knee arthroplasty revision is a challenging procedure for surgeons due to the complexity of bone loss and ligamentous failure. A primary challenge is determining the joint line (JL) height due to the existing bone loss. Restoring the physiological JL positioning is a crucial principle in TKA revision to improve range of motion, optimize extensor function, and maintain normal knee kinematics. 3

Several authors have studied methods for JL estimation using anatomical markers, such as the fibular head, the tibial tuberosity, and epicondylar distances. 3 4 It is worth noting that studies in some population groups may differ in their anatomical aspects from other ethnic groups. 5 6 7

In this context, the objective of the present study was to analyze a constant ratio between the anatomical markers of the knee, that is, the clinical medial epicondyle distance (MED), lateral epicondyle distance (LED), and condylar bicortical axis (CBA) to facilitate estimating JL positioning in TKA revision procedures in the Brazilian population.

## Materials and Methods

The Ethics Committee of Universidade Federal de São Paulo approved the present study under number CAAE 83009724.5.0000.5505. We randomly and anonymously selected 500 magnetic resonance imaging (MRI) scans of the knees of 250 women and 250 men.

The inclusion criterion was skeletally mature male or female patients.

The exclusion criteria were tests showing morphological knee bone deformity (osteophytes), implantable medical devices, and evidence of previous joint fractures.

We identified eligible patients in the institutional MRI database available on the Clinical Collaboration Platform (Carestream Health). For patients with bilateral scans, we analyzed the right knee alone.

We evaluated MED and LED at the femoral JL in coronal T2-weighted MRI and the CBA distance in axial T2-weighted MRI using the software. Measurements were in millimeters (mm).

- CBA distance: 15mm anterior to the posterior JL at the distance from the femoral mediolateral bicortical axis and the level of the two femoral epicondyles on an axial MRI scan ( Fig. 1.1 ).
- Femoral MED: Distance from the most proximal and prominent point of the crest of the clinical medial epicondyle to the distal articular point of the medial femoral condyle on a coronal MRI scan ( Fig. 1.2 ).
- Femoral LED: Distance from the most proximal and prominent point of the lateral epicondyle of the epicondylar crest to the distal articular point of the lateral femoral condyle on a coronal MRI scan ( Fig. 1.3 ).

**Fig. 1:**
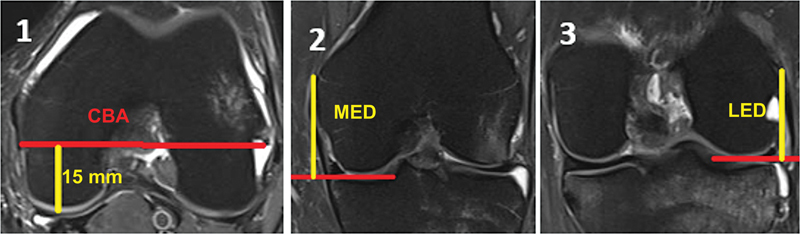
Fig. 1.1 expresses the measurement of the distance from the condylar bicortical axis (CBA); Fig. 1.2 demonstrates the medial epicondylar distance to the joint line (MED); and Fig. 1.3 demonstrates the lateral epicondylar distance to the joint line (LED).

A single knee surgeon performed all measurements twice on different days. We only computed measurements with < 10% variation in the intraobserver analysis.

Epidemiological data, including age, gender, and bone morphological distances, underwent statistical analysis for mean and standard deviation (SD) values.

The sampling methodology and sample calculations considered a 95% confidence interval (CI).

For each of the 500 pairs of LED versus CBA and MED versus CBA values, we calculated ratios for male and female subjects:



## Results

The mean age of the patients was 50.91 ± 14.76 years old. The mean age was 48.82 ± 15.43 years old for males and 53.01 ± 13.73 years old for females. The mean distance from the CBA was 72.11 ± 5.93 mm, 76.21 ± 4.83 mm in men and 68.00 ± 3.64 mm for women. The mean MED and LED were 33.39 ± 3.50 mm and 26.32 ± 4.08 mm, including 35.20 ± 3.15 mm and 27.11 ± 4.60 mm in males and 31.58 ± 2.86 mm and 25.53 ± 3.25 mm in females ( Table 1 ).

**Table 1:** Epidemiological and anthropomorphological data of knees, with number of patients, age, condylar bicortical axis, distance from the medial epicondyle to the joint line, and distance from the lateral epicondyle to the joint line

	Total (mean ± SD)	Male (mean ± SD)	Female (mean ± SD)
Patients	500	250	250
Age (years old)	50.91 ± 14.76	48.82 ± 15.43	53.01 ± 13.73
CBA (mm)	72.11 ± 5.93	76.21 ± 4.83	68.00 ± 3.64
MED (mm)	33.39 ± 3.50	35.20 ± 3.15	31.58 ± 2.86
LED (mm)	26.32 ± 4.08	27.11 ± 4.60	25.53 ± 3.25

**Abbreviations** : CBA, condylar bicortical axis; LED, distance from the lateral epicondyle to the joint line; MED, distance from the medial epicondyle to the joint line; SD, standard deviation.

For females, the lateral epicondyle ratio was 95%CI = 0.3720–0.3815 with a margin of error of ± 0.0048 and the medial epicondyle ratio was 95%CI = 0.4599–0.4707 with a margin of error of ± 0.0054. For males, the lateral epicondyle ratio was 95%CI = 0.3559–0.3671 with a margin of error of ± 0.0056 and the medial epicondyle ratio was 95%CI = 0.4568–0.4668 with a margin of error of ± 0.0050. There is evidence that the actual lateral or medial epicondyle ratio and the CBA distance are within the previously mentioned intervals with 95% confidence.

Thus, we established the following formulas for JL determination about CBA, MED, and LED:



## Discussion

The relationship between the epicondylar anatomical references and the knee biepicondylar distance for potential parameterization and determination of the femoral JL positioning was satisfactory in the Brazilian population, with adequate metric ratios in males and females.

In revision TKAs, maintaining joint height is fundamental for preserving the postoperative biomechanical functionality of the knee and ensures good joint mobility and stability. However, the technical difficulties in determining this anatomical reference are relevant due to bone loss. The surgical sacrifice of JL positioning results in a greater risk of joint biomechanical impairment and patellar wear, overload, and instability due to ligament laxity or excessive tension. 8 9 10 Modifying the JL positioning by 8 mm leads to significant functional changes and worse clinical outcomes in the postoperative follow-up. 11 12

Studies investigated the relationship between the JL and anatomical structures as a reference. These studies tried to establish intraoperative parameters to identify the JL and lateral and medial epicondyles, which are prominent and fundamental structures with a good and precise relationship between them. 3 4 However, we did not find studies in the literature determining these specific parameters for the Brazilian population.

It is worth noting that several populations, including Caucasians, Americans, Asians, and Europeans, present variable knee morphological aspects, demonstrating that anatomical features may differ in some ethnicities. 6 13 14 15 16 As such, it is essential to analyze joint parameters from mixed-race populations, such as the Brazilian population, to increase the reliability of joint morphological data. The significance of studying specific populations relies on the world's ethnic diversity and its potential influences on anatomical characteristics of the population. Thus, the present study provides Brazilian surgeons with an alternative for improving the positioning of the femoral component, especially in revision TKA, to contribute to another potential accuracy parameter for JL estimative.

The present study evaluated the relationship between JL positioning based on evident anatomical parameters (the epicondyles) in MRI scans during a revision TKA due to their greater ease of identification and measurements of epicondylar distances compared with simple radiographs. It is easy to observe the bicondylar distance during surgery; in contrast, the prominent epicondylar points may provide some difficulty in accurate identification, but it is still possible to map them intraoperatively.

Our results revealed the possibility of applying a constant multiplier to the CBA distances during TKA surgery with bone loss to estimate, from the medial, lateral, or both epicondyles, JL positioning with 95%CI, adding greater accuracy and safety to the Brazilian population. On average, MED was more proximal to LED in 27% of males and 23% of females. The existence of two parameters for epicondyle-based JL estimative can increase surgical accuracy for this determination as, depending on the anatomical features of each patient and surgical joint approach issues, one epicondyle may be more feasible for identification than the other.

Joint line positioning from the medial epicondyle is well-studied and established in the current literature, suggesting fixed distances from 23 mm to 35 mm depending on the biotypical relationships of the patients. 3 4 17 Other authors evaluated JL positioning from the fibular head. They found a relationship of 4 mm to 22 mm, demonstrating that the epicondyle is the least variable parameter when compared with the fibular head for the same purpose. 3 4 18 19 Rajagopal et al. 8 studied the proportionality relationship between the interepicondylar distance and the epicondyles to the articular surface, which was constant in the English population, concluding that this measurement can predict the JL line positioning.

The results of the present study have limitations, requiring their critical analysis by the reader. We did not record biometric profiles, that is, height and weight of the patients, to understand whether the anatomical divergences of the population may be related to other biotypical features. We did not analyze the mixed profile of the sample to determine whether the groups had race or color biases. The bone sections parameterized in the radiological study may not correspond to those performed by a surgeon depending on conditions such as bone hypoplasia or defects, a fact potentially adding bias when transposing our results for these cases.

## Conclusion

The distance from the femoral condylar bicortical axis can be a reference for determining JL positioning from the distances of the medial and lateral femoral epicondyles.
